# Quetiapine Use Is Associated with Longer ICU Stay Compared to Control and Haloperidol: A Propensity Score–Matched Analysis Using the MIMIC-IV Database

**DOI:** 10.3390/jcm14134438

**Published:** 2025-06-23

**Authors:** Ajna Hamidovic

**Affiliations:** College of Pharmacy, University of Illinois Chicago, Chicago, IL 60607, USA; ahamidov@uic.edu

**Keywords:** delirium, quetiapine, haloperidol, intensive care unit, propensity score, emulated clinical trial, Medical Information Mart for Intensive Care

## Abstract

**Background:** Due to the low certainty of existing evidence, no formal recommendation can be made for or against the use of antipsychotics over usual care in ICU patients with delirium. To advance evidence-based practice, we used observational data from the Medical Information Mart for Intensive Care (MIMIC) to estimate the effect of pre-ICU quetiapine treatment (vs. control) on the length of ICU stay. In a second, head-to-head comparison, we assessed quetiapine vs. haloperidol on the same outcome. **Methods:** We conducted two propensity score-matched procedures: 518 patients were matched based on receipt of quetiapine versus no antipsychotic (i.e., control), and 336 patients based on quetiapine versus haloperidol prior to ICU admission. After matching, we performed Bayesian generalized additive modeling (GAM) and Bayesian sensitivity analyses within a nonlinear modeling framework. **Results:** In the quetiapine versus no quetiapine analysis, the original overall covariate distance of 0.48 was reduced to 0.01 post-matching. All covariates achieved an acceptable balance, with absolute standardized mean differences below 0.1. Quetiapine use was associated with a 1.31-day longer ICU stay (posterior mean = 0.36; 95% credible interval: 0.14 to 0.59). Sensitivity analyses indicated that this effect remained robust after accounting for plausible levels of unmeasured confounding. In the quetiapine versus haloperidol analysis, the initial overall distance of 0.40 was reduced to 0.09 after matching, with all covariates similarly balanced. Compared to haloperidol, quetiapine treatment was associated with a 1.46-day longer ICU stay (posterior mean = 0.48; 95% credible interval: 0.09 to 0.88). Bayesian sensitivity analyses again indicated the robustness of the effect estimate. **Conclusions:** In these emulated clinical trials, pre-ICU treatment with quetiapine was associated with a prolonged ICU stay compared to both untreated and haloperidol conditions. Though more research in this field is needed, these findings do not support the use of quetiapine in ICU patients with delirium.

## 1. Introduction

Delirium is a serious manifestation of acute encephalopathy [1] that significantly hinders recovery in critically ill patients. It is highly prevalent in the ICU, with a meta-analysis of 16,595 patients reporting a prevalence of 31.8% [2]. Depending on age, geographic region, and ICU type, among other factors, delirium in the ICU, however, can range from 10% to 50% [3,4].

Defined in the Diagnostic and Statistical Manual of Mental Disorders, Fifth Edition [5] as a “sudden deterioration in attention, awareness, and cognition”, one form of delirium is iatrogenic in nature and caused by the intake of sedatives, opioids, and anticholinergic medications [6]. Delirium can also emerge as a manifestation of endogenous, physiological conditions, such as ischemic or hemorrhagic stroke [7], as well as disturbances originating outside of the CNS, such as sepsis [8].

The diverse underlying causes of delirium reflect its distinct pathophysiological mechanisms. Recognizing this variability is crucial in evaluating new pharmacologic treatments for delirium; yet this concept has often been overlooked. Therefore, in the present study, we focused on ICU patients with delirium arising from physiological conditions rather than drug-induced causes, to better account for this distinction.

Traditionally, the butyrophenone antipsychotic haloperidol has been the first-line treatment for ICU delirium as most clinical trials to date assessed its efficacy [9,10,11]. However, the authors of these studies concluded that there is no evidence indicating that haloperidol reduces the ICU length of stay, and they expressed low certainty concerning its effectiveness in improving other outcomes, such as 28-day mortality or mortality at the longest reported follow-up.

Since the data on the efficacy of antipsychotics other than haloperidol in ICU patients with delirium are more limited, in the present emulated clinical trial, we first evaluated the efficacy of quetiapine (vs. control) in shortening the length of ICU stay. In the second, head-to-head analysis, we compared the efficacy of quetiapine vs. haloperidol in reducing the length of ICU stay. As there is now probative evidence that haloperidol does not reduce the length of ICU stay [6], we used it in the second analysis as a comparator to assess whether quetiapine has a differential effect on this outcome. We focused on the length of ICU stay because a large meta-analysis of forty-one studies showed that patients with delirium had ICU stays that were, on average, 4.77 days longer than those without delirium [12]. Delirium not only increases the health risks associated with a prolonged ICU stay but also elevates daily care costs to an average of USD 600 per day, primarily due to factors related to increased monitoring and additional interventions [13].

## 2. Materials and Methods

### 2.1. Study Design and Data Source

Implementing a large-sample retrospective analysis, we first assessed the length of ICU stay in hospitalized patients with delirium receiving quetiapine (relative to control patients) prior to ICU admission. Using the same procedure, we then assessed the length of ICU stay in patients receiving quetiapine vs. haloperidol.

We obtained the data from the latest MIMIC-IV database (version 3.1), which contains electronic health records from over 65,000 patients admitted to the ICU at the Beth Israel Deaconess Medical Center (Boston, MA, USA) from 2008 to 2022. Deidentified data are available on PhysioNet upon signing Data Use Agreements and completing the required training. We merged data from different modules based on the patients’ ID and hospital stay. The Beth Israel Deaconess Medical Center and Massachusetts Institute of Technology approved the release of the deidentified and publicly available data without a requirement for individual patient informed consent.

### 2.2. Patients, Exposure, Outcome, and Covariates

We extracted data of the critically ill adults (age ≥ 18 years) with delirium who were admitted to the ICU. The following ICD codes were extracted for the purpose of defining delirium in the present study: delirium due to known physiological condition, vascular dementia with delirium, subacute delirium, and senile dementia with delirium. We analyzed the first admission data in the event a patient was admitted multiple times. We excluded patients who received any other typical or atypical antipsychotic agent. Hence, in the first analysis, patients administered any second-generation antipsychotic were excluded; in the second analysis, patients administered any antipsychotic other than quetiapine or haloperidol were excluded. 

Since causality requires temporal ordering, the administration of quetiapine/haloperidol had to occur during hospitalization but prior to the ICU admission. We coded control/quetiapine as 0/1 and haloperidol/quetiapine as 0/1 in the two analyses, respectively. The study outcome measure was the length of stay in the ICU. We collected the following factor (0 = no; 1 = yes) variables: antidepressant administration, benzodiazepine administration, cancer, chronic obstructive pulmonary disease, depression, diabetes, hypertension, myocardial infarction, opioid administration, sepsis, and stroke. Additionally, we collected age and gender.

### 2.3. Statistical Analysis

We performed propensity score matching using the MatchIt [14] package in R version 4.3.2 to address potential confounding and achieve covariate balance: first, between the treatment (quetiapine) and control (no antipsychotic) groups; then, between quetiapine and haloperidol groups. For both propensity matching procedures, we estimated propensity scores using a logistic regression model, with the treatment variable as the outcome and the following covariates: age, antidepressant administration, benzodiazepine administration, cancer, chronic obstructive pulmonary disease, depression, diabetes, gender, hypertension, myocardial infarction, opioid administration, sepsis, and stroke. To restrict matches within a reasonable distance, we used the nearest neighbor matching without replacement at a 1:1 ratio and a caliper of 0.2 standard deviations of the logit of the propensity score. We assessed model validity by calculating standardized mean differences (SMDs), with SMD < 0.1 indicative of a balanced distribution of confounding factors between groups.

Using the brms [15] package in R and adjusting for all covariates used in propensity score matching, we conducted Bayesian generalized additive modeling (GAM) to assess the association between quetiapine exposure and length of ICU stay. We repeated the same model for the second GAM assessing the relationship between haloperidol vs. quetiapine and the length of ICU stay. We centered and standardized age prior to fitting it in the GAM models, following which we included it as a smooth term to flexibly capture potential nonlinear effects. We specified informative priors to regularize estimates as normal (0, 1) for linear coefficients and student_t (3, 0, 2) for the smoothness parameters of the spline term. We fitted the model with 4 Markov Chain Monte Carlo (MCMC) chains, each with 4000 iterations (1000 warmup). To improve sampler efficiency and convergence, we set the adapt_delta parameter to 0.995. We assessed convergence using the potential scale reduction factor R̂ and summarized posterior distributions using means and 95% credible intervals. We interpreted nonlinear effects of age based on the estimated smooth function and its credible intervals.

In the last step, we conducted a sensitivity analyses to assess the potential impact of unmeasured confounding on the estimated effect of treatment on length of stay. We implemented Bayesian nonlinear regression with an inverse Gaussian family and a log link. We incorporated an explicit bias term in a Bayesian nonlinear regression to account for potential unobserved confounding. We modeled the nonlinear formula as

length of stay = exp (β_0_+ β_quetiapine_ × quetiapine + bias)

where β_0_, β_quetiapine_, and bias were estimated intercept terms. To evaluate the robustness of our findings to unobserved confounding, we specified a prior on the bias parameter as ∼N (0, 2.5). We repeated the sensitivity analysis twice—first, following the first analysis (quetiapine vs. control), and again, following the second analysis (quetiapine versus haloperidol).

## 3. Results

### 3.1. Participant Flow Diagrams

**Figure 1:**
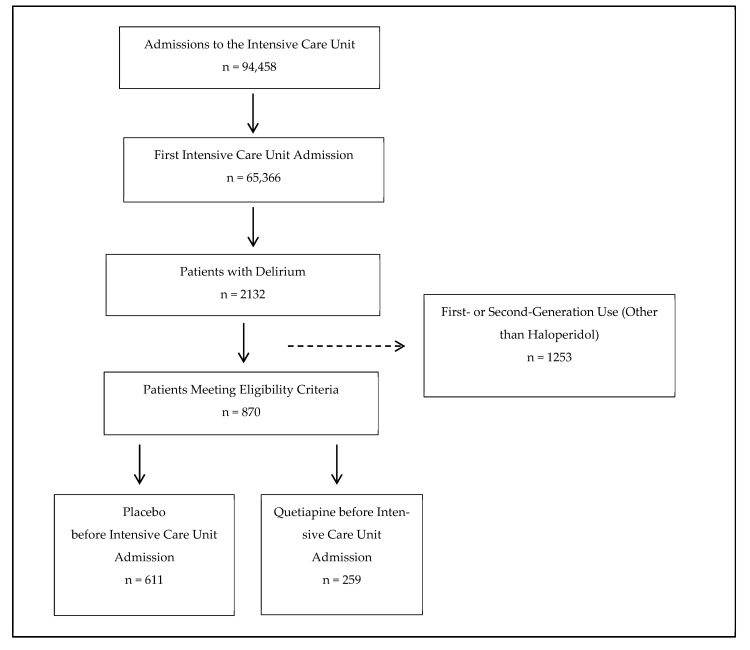
Participant flow diagram.

Of the 94,458 total ICU visits, 65,366 were first ICU visits, of which there were 2132 patients with delirium. After the exclusion of patients who were administered antipsychotics other than quetiapine, 870 met the eligibility criteria. Of these, 259 received quetiapine (Figure 1). The participant flow diagram for the second, quetiapine vs. haloperidol, analysis is identical other than the number patients meeting the inclusion criteria—259 who received quetiapine and 168 who received haloperidol.

### 3.2. Propensity Score Matching—Quetiapine Versus Control

**Figure 2:**
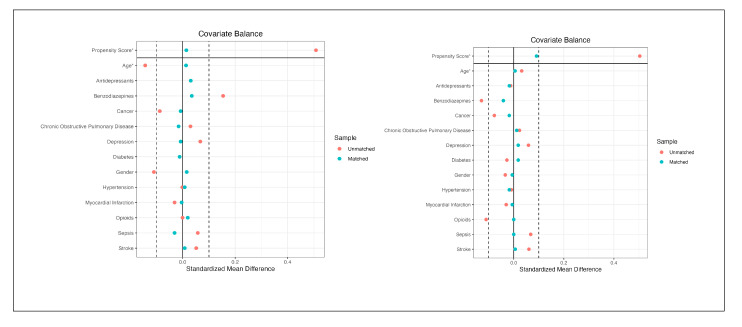
Covariate balance in the unmatched and matched treatment groups in the (**left**) quetiapine/control sample and (**right**) quetiapine/haloperidol sample. (* continuous covariate).

After matching, the total of 259 individuals in the quetiapine group were matched to 259 individuals in the control group, resulting in a matched sample of 518 individuals. Prior to matching, the overall distance between the groups was 0.4888. Following the matching procedure, the overall distance was substantially reduced to 0.0135. All the covariates achieved an adequate balance of absolute SMDs below 0.1 (see Figure 2-left and Table 1).

### 3.3. Propensity Score Matching—Quetiapine Versus Haloperidol

The matching procedure of the 168 patients in the quetiapine group to the 168 patients in the haloperidol group resulted in a matched sample size of 336. Prior to matching, the overall distance between the groups was 0.4988, which was reduced to 0.0901. All the covariates achieved an adequate balance of absolute SMDs below 0.1 (see Figure 2-right and Table 1).

### 3.4. Generalized Additive Modeling—Quetiapine Versus Control

Quetiapine administration resulted in an increased length of stay (posterior mean = 0.36, 95% credible interval [CrI]: 0.14 to 0.59). Holding all variables constant, quetiapine increased the length of stay by 43%. That is, the baseline length of stay of 3.03 days was extended to 4.34 days (1.31 days) by quetiapine. A longer length of stay was also observed in patients with stroke (posterior mean = 0.35, 95% CrI: 0.08 to 0.65), sepsis (posterior mean = 0.32, 95% CrI: 0.06 to 0.60), and those receiving benzodiazepines (posterior mean = 0.42, 95% CrI: 0.20 to 0.65) or opioids (posterior mean = 0.24, 95% CrI: 0.01 to 0.46). There was no strong evidence of an increased length of stay in patients with cancer, COPD, myocardial infarction, or other comorbidities, as the CrIs for these estimates included zero. The relationship between age and the length of stay was nonlinear, as indicated by the smoothing spline hyperparameter (posterior mean = 0.73, 95% CrI: 0.04 to 2.23). All chains converged successfully with R̂ values of 1.00 for all parameters, indicating good mixing. Effective sample sizes (bulk effective sample size and tail effective sample size) were high across all parameters, suggesting a reliable estimation of posterior distributions. See Table 2 for details.

### 3.5. Generalized Additive Modeling—Quetiapine Versus Haloperidol

Quetiapine administration was associated with an increased length of stay (posterior mean = 0.48, 95% credible interval [CrI]: 0.09 to 0.88). Holding all variables constant, quetiapine increased the length of stay by 62%. That is, the baseline length of stay of 2.36 days was extended to 3.82 days (1.46 days) by quetiapine. A longer length of stay was also observed in patients with sepsis (posterior mean = 0.60, 95% CrI: 0.09 to 1.24) and those receiving benzodiazepines (posterior mean = 0.46, 95% CrI: 0.03 to 0.91). There was some indication of longer stays in patients with stroke (posterior mean = 0.44, 95% CrI: −0.10 to 1.08) and opioid use (posterior mean = 0.38, 95% CrI: −0.13 to 0.83), but the credible intervals included zero, indicating uncertainty. No strong evidence for an increased length of stay was found in patients with cancer, COPD, myocardial infarction, or other comorbidities, as their CrIs included zero. The relationship between age and length of stay was modeled nonlinearly with a smoothing spline hyperparameter estimate (posterior mean = 1.22, 95% CrI: 0.10 to 3.29), reflecting flexible age effects. All chains converged successfully with R̂ values of 1.00 for all parameters, indicating good mixing. Effective sample sizes (bulk and tail ESS) were high across parameters, suggesting a reliable posterior estimation. See Table 2 for details.

### 3.6. Sensitivity Analysis—Quetiapine Versus Control

The posterior estimate for the bias parameter was close to zero (estimate = 0.012), with a wide 95% credible interval ranging from –0.95 to 1.00. Hence, any bias from unmeasured confounding appears to be minimal under the assumed moderate confounding scenario. The R̂ value was 1.00, indicating good convergence, and the bulk and tail effective sample sizes were 9520 and 10,875, respectively, suggesting a reliable posterior estimation. In summary, the findings suggest that the estimated effect of quetiapine is relatively robust to plausible levels of unknown confounding.

### 3.7. Sensitivity Analysis—Quetiapine Versus Haloperidol

The posterior estimate for the bias parameter was close to zero (estimate = 0.005), with a wide 95% credible interval ranging from –0.97 to 0.98. This suggests little evidence of substantial bias due to unmeasured confounding. The R̂ value was 1.00, indicating good convergence, and the bulk and tail effective sample sizes were 11,177 and 12,283, respectively, supporting reliable posterior estimation.

## 4. Discussion

The present results indicate a significantly longer ICU stay in patients who received quetiapine relative to the control or patients who received haloperidol. These findings may guide more judicious selection of antipsychotic medications in the ICU, potentially reducing unnecessary risks and costs resulting from prolonged ICU stays.

The working group of the current delirium treatment guideline from Society of Critical Care Medicine [6] concluded that there is insufficient evidence to either support or refute the use of antipsychotic agents in ICU patients with delirium. This recommendation is based on the general lack of positive results from rigorous haloperidol clinical trials and the absence of well-powered studies involving other antipsychotic agents. For example, investigators of the double-blind, placebo-controlled, randomized Modifying the Incidence of Delirium (MIND) clinical trial [11] administered haloperidol to medical/surgical ICU patients with delirium across six tertiary care medical centers in the United States. They reported no significant difference in the ICU length of stay (a secondary outcome) between the 179 patients who received the placebo and the 192 who received haloperidol. Similarly, in the large Agents Intervening against Delirium in Intensive Care Unit (AID-ICU) trial, there was no statistically significant difference in the length of hospital stay (a primary outcome) between 501 patients receiving haloperidol and 472 patients receiving placebo. This was a this randomized, double-blind fashion conducted across 18 general intensive care units (ICUs) in Europe [9]. Examining quetiapine, a retrospective study involving 47 ICU patients in Saudi Arabia reported no difference in the ICU length of stay among patients treated with quetiapine, haloperidol, risperidone, or olanzapine [16]—a finding similar to a prospective clinical trial conducted in Thailand involving 52 patients who received either quetiapine or haloperidol [17]. Conversely, Zakhary et al. [18] reported a reduced ICU stay in 100 patients in Egypt randomized to haloperidol or a placebo in a double-blind randomized fashion. The results of the study reported here differ from these three studies evaluating quetiapine vs. haloperidol as we found that quetiapine extends the stay in the ICU relative to both haloperidol and control groups.

Although delirium involves dysfunction across multiple neurotransmitter systems, anticholinergic activity appears to play a particularly prominent role. This is suggested by the central cholinergic deficit hypothesis of delirium [19] and supported by the finding of a greater likelihood for developing in-hospital delirium in patients with low pre-admission anticholinesterase levels [20]. Further support comes from a study of 278 inpatients with delirium in whom exposure to medications with anticholinergic properties was independently associated with a subsequent increase in delirium symptom severity [21]. Notably, this association remained significant after adjusting for the initial severity of delirium, presence of comorbid conditions, as well as medications, suggesting that delirium is specific to the intake of medications with pronounced anticholinergic effects. These findings appear to extend to multiple settings and populations. When implementing an anticholinergic rating system where a score of 3 indicates a medication’s established clinically relevant anticholinergic impact on delirium, quetiapine was scored 3 and found to be the top definitive anticholinergic drug in European patients treated in specialized geriatric units [22], and among the five most commonly prescribed medications to treat hospice-related delirium [23].

Quetiapine as the parent drug generally has negligible to very low affinity for muscarinic cholinergic receptors. It undergoes extensive metabolism, primarily by CYP3A4, with less than 5% excreted unchanged. In contrast to quetiapine, its active metabolite, norquetiapine, acts as a strong antagonist at the muscarinic M1, M3, and M5 receptors [24]. This property contributes to a greater anticholinergic burden of quetiapine (e.g., sedation, dry mouth, and cognitive impairment) relative to haloperidol, risperidone, and aripiprazole, which have a minimal muscarinic receptor affinity [25,26]. Norquetiapine is also further metabolized, and its elimination half-life is longer than that of quetiapine. Genetic variations in enzymes like CYP2D6 can impact the clearance of norquetiapine and potentially influence the risk of adverse effects [27]. This, along with a strong antihistaminergic (H1-blocking) activity by quetiapine that causes sedation, may represent a mechanism of the quetiapine’s negative effect on ICU discharge in patients with delirium reported in the present study.

It should be noted that the present findings apply to a subgroup of patients with delirium due to a known physiological condition, vascular dementia, subacute delirium, or senile dementia with delirium. Delirium can also manifest from substance withdrawal or acute effects of medications, which may account for up to 39% of delirium cases [28]. Hence, our study is limited in generalizability, which presents a limitation. Furthermore, though selection bias can be minimized in emulated clinical trials such as the ones presented here, it cannot be entirely eliminated. To deal with this, we implemented explicit inclusion/exclusion criteria that would be used in a hypothetical randomized controlled trial, thereby reducing arbitrary or biased selection. These study limitations should be evaluated against the strengths of the present study, such as a relatively large sample size, comparison of quetiapine to both control as well as haloperidol conditions, successful propensity score matching, and robustness of study findings as indicated by the results of our sensitivity analysis.

Further research in this field that includes additional outcomes, such as the effect of quetiapine on the length of delirium, is critical. We reached the decision to assess the length of ICU stay as this is a highly relevant, resource-intensive, and patient-centered measure [13]. Critically, focusing on one highly relevant outcome is of methodological and statistical relevance. Our propensity score matching strategy was optimized specifically to reduce confounding for the outcome of ICU stay. Including additional outcomes would require a separate set of confounders and possibly a different matching strategy, thereby diluting the causal inference of any one outcome. In addition, including additional outcomes in our Bayesian generalized model would have likely burdened the model with multiple response distributions; hence, focusing the analysis to the ICU stay allows for a full interpretive focus on posterior estimates, credible intervals, and model diagnostics. Critically, our Bayesian sensitivity analysis to unmeasured confounding was conducted with a clearly defined causal pathway, and with a single outcome, it is easier to specify plausible bias scenarios and explain robustness. This is particularly important when using observational data such as that from MIMIC. Finally, adding more outcomes would require multiple testing adjustments and hierarchical outcome modeling, which hold the potential to reduce the power and dilute clinically meaningful signals. In summary, the focus on the single ICU length of stay outcome preserves methodological rigor, simplifies model assumptions, provides a clear conclusion, and avoids pitfalls of outcome multiplicity in a non-randomized emulation framework.

Furthermore, in alignment with the intention-to-treat principle, we defined exposure as the initiation of a treatment strategy—quetiapine, haloperidol, or no antipsychotic—rather than the specific dose prescribed. This binary approach (medication receipt vs. no receipt) helps preserve interpretability, reduce confounding complexity, and avoid model overfitting. Future studies focused specifically on dose–response effects would be well-suited to explore the implications of dosing variation. Another limitation is the lack of information on delirium motor subtypes—specifically, hypoactive and hyperactive delirium—in the MIMIC database. These subtypes differ in clinical presentation, prognosis, and potentially in response to pharmacologic treatment. Because MIMIC does not consistently capture structured data or validated assessments differentiating delirium subtypes, we were unable to stratify or adjust analyses based on this important clinical feature. This limitation may obscure differential effects of antipsychotics across delirium phenotypes and warrants further investigation in prospective datasets with richer delirium characterization.

In conclusion, the present findings do not support the administration of quetiapine to patients with delirium that is due to an underlying physiological condition. A relatively strong anticholinergic and antihistaminic profile of quetiapine may adversely affect the course of the ICU stay, thereby exposing patients to risks and costs associated with spending more time in the ICU.

## Figures and Tables

**Table 1:** Standardized mean difference (SMD) according to treatment group after propensity score matching.

Quetiapine vs. Control Comparison				Quetiapine vs. Haloperidol Comparison		
Variable	Means		SMD	Means		SMD
	Quetiapine (n = 259)	Control (n = 259)		Quetiapine (n = 168)	Haloperidol (n = 168)	
Propensity Score	0.3375	0.3361	0.0135	0.5825	0.5723	0.0901
Age	71.7992	71.6178	0.0125	71.4167	71.3393	0.0053
Antidepressants	0.2317	0.2008	0.0732	0.2262	0.2440	−0.0423
Benzodiazepines	0.5676	0.5328	0.0701	0.5238	0.5655	−0.0840
Cancer	0.2201	0.2278	−0.0186	0.2798	0.2976	−0.0431
Chronic Obstructive Pulmonary Disease	0.1776	0.1931	−0.0404	0.1667	0.1548	0.0311
Depression	0.2432	0.2510	−0.0180	0.2024	0.1845	0.0416
Diabetes	0.3475	0.3591	−0.0243	0.3929	0.3750	0.0375
Gender	0.3707	0.3552	0.0320	0.3988	0.4048	−0.0123
Hypertension	0.4788	0.4710	0.0155	0.4702	0.4881	−0.0357
Myocardial Infarction	0.2317	0.2355	−0.0092	0.2560	0.2619	−0.0141
Opioids	0.6873	0.6680	0.0416	0.7976	0.7976	0.0000
Sepsis	0.2819	0.3127	−0.0687	0.2143	0.2143	0.0000
Stroke	0.2510	0.2432	0.0178	0.1964	0.1905	0.0137

**Table 2:** Parametric and smooth coefficients from the general additive model.

Quetiapine vs. Control							Quetiapine vs. Haloperidol							
	Estimate	Estimated Error	Lower 95% CI	Upper 95% CI	R^	Bulk Effective Sample Size	Tail Effective Sample Size	Estimate	Estimated Error	Lower 95% CI	Upper 95% CI	R^	Bulk Effective Sample Size	Tail Effective Sample Size
(Intercept)	1.1054	0.1517	0.8202	1.4135	1.0002	15,050.8	9183.7	0.8707	0.3535	0.2448	1.6304	1.0004	9304.9	7770.6
Age	−0.2962	0.8889	−1.8913	1.5408	1.0001	6712.4	8406.9	−0.4389	0.9614	−2.2499	1.4932	1.0004	9733.1	8959.6
Antidepressants	−0.0262	0.1395	−0.2804	0.2655	1.0009	11,642.8	7625.1	−0.1457	0.2733	−0.6314	0.4252	1.0000	9854.7	7752.8
Benzodiazepines	0.4224	0.1145	0.2012	0.6508	1.0002	13,847.2	9062.8	0.4643	0.2204	0.0326	0.9068	1.0006	8155.2	7514.7
Cancer	0.0529	0.1370	−0.2045	0.3298	1.0004	13,744.6	8865.3	−0.0630	0.2152	−0.4622	0.3814	1.0003	10,877.2	8611.4
Chronic Obstructive Pulmonary Disease	0.0522	0.1465	−0.2170	0.3567	1.0000	14,398.4	8627.2	0.1554	0.3290	−0.3888	0.9077	1.0007	10,220.0	6952.2
Depression	−0.0450	0.1285	−0.2868	0.2122	1.0001	12,973.0	9017.5	0.2824	0.3229	−0.2866	0.9845	1.0001	8208.7	7287.8
Diabetes	−0.0921	0.1246	−0.3332	0.1600	1.0001	11,529.1	8523.1	0.2103	0.2291	−0.2175	0.6871	1.0003	10,038.8	8217.5
Gender	−0.0257	0.1179	−0.2536	0.2125	1.0006	11,947.3	8742.4	0.2107	0.2209	−0.2077	0.6638	1.0008	9340.2	8545.4
Hypertension	0.0732	0.1126	−0.1480	0.2969	1.0007	13,561.5	8861.5	0.0373	0.2186	−0.3919	0.4767	1.0001	10,571.8	8620.8
Myocardial Infarction	0.0293	0.1453	−0.2491	0.3259	1.0001	11,403.8	9372.3	0.0575	0.2641	−0.4310	0.6068	1.0001	9124.3	7706.8
Opioids	0.2412	0.1148	0.0092	0.4630	1.0001	12,971.3	8317.5	0.3800	0.2438	−0.1260	0.8311	1.0003	10,288.7	7068.3
Quetiapine	0.3598	0.1135	0.1394	0.5881	1.0006	13,568.9	8805.8	0.4766	0.2037	0.0857	0.8790	1.0001	10935.9	8509.2
Sepsis	0.3174	0.1352	0.0630	0.5982	1.0003	13,171.7	8420.9	0.6023	0.2917	0.0891	1.2361	1.0008	9835.7	7022.8
Stroke	0.3502	0.1459	0.0781	0.6549	1.0004	10,931.9	7207.3	0.4404	0.3018	−0.0984	1.0841	1.0006	8998.3	7678.7

## Data Availability

Restrictions apply to the availability of these data. Data were obtained from physionet.org (accessed on 2 May 2025) and are available from physionet.org with the permission of physionet.org.
